# Drug repositioning: A bibliometric analysis

**DOI:** 10.3389/fphar.2022.974849

**Published:** 2022-09-26

**Authors:** Guojun Sun, Dashun Dong, Zuojun Dong, Qian Zhang, Hui Fang, Chaojun Wang, Shaoya Zhang, Shuaijun Wu, Yichen Dong, Yuehua Wan

**Affiliations:** ^1^ Institute of Pharmaceutical Preparations, Department of Pharmacy, Zhejiang University of Technology, Hangzhou, China; ^2^ Institute of Information Resource, Zhejiang University of Technology, Hangzhou, China; ^3^ Hangzhou Aeronautical Sanatorium for Special Service of Chinese Air Force, Hangzhou, China; ^4^ Faculty of Chinese Medicine, Macau University of Science and Technology, Macau, China

**Keywords:** drug repurposing, bibliometrics, drug development, COVID-19, virtual screening

## Abstract

Drug repurposing has become an effective approach to drug discovery, as it offers a new way to explore drugs. Based on the Science Citation Index Expanded (SCI-E) and Social Sciences Citation Index (SSCI) databases of the Web of Science core collection, this study presents a bibliometric analysis of drug repurposing publications from 2010 to 2020. Data were cleaned, mined, and visualized using Derwent Data Analyzer (DDA) software. An overview of the history and development trend of the number of publications, major journals, major countries, major institutions, author keywords, major contributors, and major research fields is provided. There were 2,978 publications included in the study. The findings show that the United States leads in this area of research, followed by China, the United Kingdom, and India. The Chinese Academy of Science published the most research studies, and NIH ranked first on the h-index. The Icahn School of Medicine at Mt Sinai leads in the average number of citations per study. Sci Rep, Drug Discov. Today, and Brief. Bioinform. are the three most productive journals evaluated from three separate perspectives, and pharmacology and pharmacy are unquestionably the most commonly used subject categories. Cheng, FX; Mucke, HAM; and Butte, AJ are the top 20 most prolific and influential authors. Keyword analysis shows that in recent years, most research has focused on drug discovery/drug development, COVID-19/SARS-CoV-2/coronavirus, molecular docking, virtual screening, cancer, and other research areas. The hotspots have changed in recent years, with COVID-19/SARS-CoV-2/coronavirus being the most popular topic for current drug repurposing research.

## 1 Introduction

Sir James Black, a winner of the 1988 Nobel Prize, clearly recognized well before the 21st century that drug repurposing strategies would occupy an important place in the future of new drug discovery (Raju, 2000). In 2004, Ted T. Ashburn et al. (Ashburn and Thor, 2004) summarized previous research and developed a general approach to drug development using drug repurposing, retrospectively looking for new indications for approved drugs and molecules that are waiting for approval for new pathways of action and targets. These molecules are usually safe in clinical trials but do not show sufficient efficacy for the treatment of the disease originally targeted (Southan et al., 2013). The definition of the term “drug repurposing” has been endorsed by scholars (Dudley et al., 2011) and used by them (Li et al., 2011; Cheng et al., 2012). It should be pointed out that the synonyms of “drug repurposing” often used by academics also include drug repositioning (Rosa and Santos, 2020), drug rediscovery (Simsek et al., 2018), drug redirecting (Jang et al., 2019), drug retasking (Scherman and Fetro, 2020), and therapeutic switching (Kim et al., 2019; Kurdi et al., 2019). After the research study by Ashburn et al., Allarakhia et al. expanded the starting materials for drug repositioning to include products that were discontinued for commercial reasons, expired patents, and candidates for laboratory testing (Allarakhia, 2013). In the discovery process of a completely new drug, the difficulty usually lies in its safety and efficacy, which are the main potential causes of failure of most drugs in the approval (Schuster et al., 2005) or clinical development stage (Milne, 2017). Using existing knowledge about a drug or known target (Mercorelli et al., 2018), the time, risk, and cost of developing a drug using drug repositioning are reduced (Joshua, 2011), thereby greatly increasing the efficiency and economics of drug development, providing a better risk–reward trade-off, and making it easier to win the favor of venture capital firms (Ashburn and Thor, 2004).

Since the 1990s, the repositioning of sildenafil for male erectile dysfunction (Goldstein et al., 1998) and pulmonary hypertension (Badesch et al., 2007), the development of a new efficacy of bupropion for smoking cessation (Hurt et al., 1997), new applications of thalidomide for multiple myeloma (Singhal et al., 1999; Barlogie, 2001), and chronic graft-versus-host disease (Vogelsang et al., 1992) have generated intense interest from pharmaceutical companies and academics (Kumar et al., 2019). These classic success stories rely on three traditional approaches: 1) molecular biology approaches (Pujol et al., 2010), 2) *in vivo* and *ex vivo* experimental approaches (Kuter, 2007; Swinney and Anthony, 2011), and 3) expert knowledge-based approaches (Kumar et al., 2019). Due to the unknown, complex, and information-fragmented nature of drug candidates and potential new mechanisms of action (Yella et al., 2018), this activity is dependent on multiple factors, and success is often fortuitous (Kumar et al., 2019). At the beginning of the 21^st^ century, cheminformatics (Feng et al., 2007; Joshua, 2011), bioinformatics (Salazar et al., 2006; Feng et al., 2021), systems biology (Lv et al., 2018; Turanli et al., 2021), genomics (Zhao et al., 2016; Mirza et al., 2017), polypharmacology (Reddy and Zhang, 2013; Anighohro et al., 2014), precision medicine (Delavan et al., 2018; Tanoli et al., 2020), and other disciplines, combined with artificial intelligence (Yang et al., 2019), have developed rapidly. These rapidly growing disciplines have promoted the generation of systematic (Talevi and Bellera, 2020) computer methods to make the drug repositioning process cheaper and shorter (Vanhaelen et al., 2017; Luo et al., 2021). Computational drug repositioning is classified as “disease-centric” or “target/gene-centric” or “drug-centric” depending on the source of discovery (Li et al., 2016). This process relies on public biochemical databases such as DrugBank (Mihai et al., 2019; Mazzolari et al., 2020), ChEMBL (Mendez et al., 2019), Cmap (Lin et al., 2020), PDB (Berman et al., 2000), OMIM (Amberger et al., 2014), etc., to provide the appropriate information. In fact, to make the computational drug repurposing process, including the molecular docking and virtual screening steps, more convenient, database tools specifically developed for drug repurposing, such as EK-DRD (Zhao et al., 2019), DREIMT (Troulé et al., 2021), DrugSig (Wu H. et al., 2017), RepoDB (Malas et al., 2019), Promiscuous 2.0 (Gallo et al., 2021), etc., have been reported in the last few years. In addition, it has been found in the literature that only 10% of the research results have been carried out in the “drug-centric” pathway, which holds great prospects for future development (Parisi et al., 2020). With the help of database tools, it is now possible to perform computational screening of even a staggering number of hundreds of millions of compounds (Fischer et al., 2020). Computer methods to carry out this screening include machine learning (Napolitano et al., 2013), network modeling (Francisco, 2013; Lotfi Shahreza et al., 2018), text mining, and semantic reasoning (Christos et al., 2011; Yuan et al., 2017; Ji et al., 2020), among others. The ultimate objective of repositioning is to transfer one or two of the most relevant results to clinical applications. Therefore, validation is quite important (Li et al., 2016) and requires consideration of multiple factors, such as price, toxicity levels, bioavailability, and differences between validated and computational models (Li et al., 2016; Jarada et al., 2020). Current validation methods include experimental validation (Kang et al., 2014), electronic health records to aid validation (Xu et al., 2015), cross-validation (Wu Z. et al., 2017; Ozsoy et al., 2018), gold standard dataset evaluation (Luo et al., 2021), literature citation validation (Chopra et al., 2016), and expert consultation (Jarada et al., 2020).

Today, drug repositioning is increasingly prominent in the development of drugs for a variety of neurological diseases (Athauda and Foltynie, 2018; Kessing et al., 2019), cancer (Gupta et al., 2013; Efferth, 2017), rare diseases (Sardana et al., 2011; Southall et al., 2019), and infectious diseases (Pietschmann, 2017; Muratov et al., 2021). An increasing number of pharmaceutical companies are also establishing relevant R&D programs (Kettle and Wilson, 2016) or funding support (Tummino et al., 2021). To translate relevant research results efficiently and smoothly, national departments within the United Kingdom, the United States, and the Netherlands have (Paul and Lewis-Hall, 2013; Vanhaelen et al., 2017) launched initiatives or programs to build partnerships between pharmaceutical companies and academia and to further explore scientific and commercial opportunities (Yella et al., 2018). It is certain that drug repositioning currently presents several dilemmas, such as intellectual property challenges (Breckenridge and Jacob, 2019), data platforms, and analytical techniques that need to be improved (Kumar et al., 2019), that financial support remains important for technology development and clinical trials (Verbaanderd et al., 2021), and that some scientists deny the practical utility of the approach (Edwards, 2020).

There have been systematic analyses of terminology in the drug repurposing literature (Langedijk et al., 2015), text mining of drug–disease combinations (Baker et al., 2018), and the progression of a particular drug (Li X. et al., 2020), but no studies have yet provided a broad overview of publications on the topic of drug repurposing research. When independent researchers or collectives (including pharmaceutical companies, academia, and government departments) seek drug repurposing partnership partners and seek to obtain a concise overview of comprehensive current research hotspots, the lack of relevant intelligence analysis to aid decision-making often makes the process convoluted and time-consuming (Frail et al., 2015). The bibliometric approach can solve the aforementioned problems relatively fairly, but at present, scholars have only studied the bibliometrics of aspirin, a drug repurposing (Li X. et al., 2020); there has not been a panoramic study of drug repurposing, and therefore, this study is necessary. Bibliometrics is a useful tool combining multiple parameters for the quantitative analysis of scholarly publications and is currently used to assess research hotspots and trends in a wide range of disciplines and industries, such as management (Vogel and Güttel, 2013; Feng et al., 2017), sociology (Rey-Martí et al., 2016; Sharifi, 2021), economics (Zhang et al., 2019), medicine (Tao et al., 2012; Powell et al., 2016), environmental engineering (Colares et al., 2020; Mao et al., 2021), and agronomy (Canas-Guerrero et al., 2013; Giraldo et al., 2019). Therefore, this study uses bibliometric methods (Leung et al., 2017) to quantitatively assess the following elements of drug repositioning publications: 1) major contributors: countries, research institutions, and authors; 2) modes of collaboration: intercountry collaborations; 3) the most productive journals; 4) the most frequently used disciplinary knowledge; and 5) research trends, judged by analyzing author keywords, Essential Science Indicators (ESI) high citations, and hot research studies.

## 2 Methodology and data processing

### 2.1 Data collection

We use the Web of Science™ core database, an authoritative academic information data service platform produced by Clarivate (version ^©^ 2021 Clarivate.). Due to its rigorous selection of journals, the Web of Science (WOS) Core Collection Database is now internationally recognized as a database for evaluating the scientific output or disciplinary development of scholars and institutions. Among the subdatabases, SCI-E mainly includes global journals in basic science research, covering basic pharmacological and medical research related to the theme of this study, “drug repositioning,” while SSCI includes social science, covering ethical, nursing, psychological, and other social science research related to this study.

**FIGURE 1:**
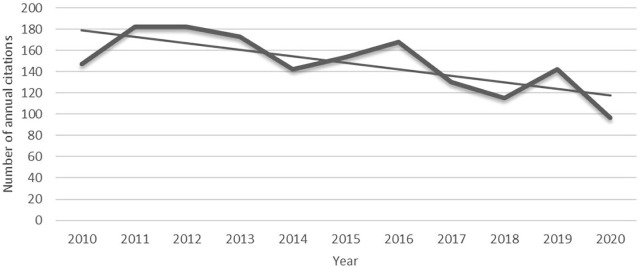
Total number of citations per year from 2010 to 2020 for 30 publications published from 1990 to 2009.

The data were obtained on 25 October 2021 through the WOS Core Collection Database Citation Indexes SCI-E and SSCI, using the formula “drug repurposing” OR “drug repositioning” OR “drug rediscovery” OR “therapeutic switching” OR “drug redirecting” OR “drug rediscovery” OR “drug retasking” search query, searching in the “subject” field and defining the document type as “Article” and “Review”. The publication time parameters were initially limited to publications related to “drug repositioning” published between 1990 and 2020. A total of 3,009 documents were obtained, of which only 31 were published in two decades from 1990 to 2009. Of these 31 documents, except for one document that is still frequently used by scholars as a retrospective source for drug repurposing definitions in these years (Ashburn and Thor, 2004), the remaining 30 were cited by other authors during the period of 2010–2020 as shown in Figure 1. The overall level of interest in these studies shows a fluctuating downward trend as opposed to the rising citation fervor for drug repurposing, entering a stage of decline even under the less-demanding evaluation criterion of a 5-year maturation window (Jacsó, 2009). As the literature ages, its content becomes stale and obsolete in the perspective of intelligence sources, and the value of the metrics for judging current research trends is low. Therefore, we further narrowed the study to 2,978 publications published from 2010 to 2020.

### 2.2 Data import and deduplication

The complete records of all retrieved documents are downloaded and imported for processing into Derwent Data Analyzer (DDA) version V10, a data cleaning, multiperspective data mining, and visualization software from Clarivate that improves data analysis efficiency and reduces labor costs. After importing all records of WOS documents into DDA, they are classified and measured according to a list of fields such as keyword, country/region, institution, author, research field, journal, etc. For each item in the list fieldset, DDA has a built-in data cleaning tool for automatic data deduplication.

### 2.3 Data splitting or merging

After the machine has removed duplicates, the items in the set of fields still need to be manually verified for splitting or merging. It is to be noted that the regions of certain countries are presented separately, while they are usually considered as a single country internationally. Therefore, we need to perform merging, such as combining Wales, Scotland, England, and Northern Ireland into the United Kingdom column and combining Hong Kong and Macau regions into the China column.

To address the possible problem of different authors with the same name, the following two main verification steps were performed: 1) returning to the WOS database to search for publications under that author’s name under the original search formula conditions and 2) for authors whose publications provide disputed information (this also includes three cases: first, two or more authors with the same name but not the same person; second, two or more authors with the same abbreviated name, but the full names were found to be different after a search; and third, similar signatures being different variants of the same author’s name), in addition to searching the ORCID-related information of the authors concerned for judgment, we checked different institutional websites as well as encyclopedias to look for changes in the study and work history of authors with the same or similar names from 2010 to 2020 to determine whether they were the same person. Based on the verification, we then split or merged the results.

### 2.4 Data analysis and visualization

After data cleaning and matrix analysis by DDA, various types of cluster plots and bubble plots can be obtained to reveal the useful information behind the data. The bibliometric fields of publication volumes, countries, international collaborations, institutions, research areas, journals, authors, highly cited research studies, and author keywords were analyzed in this study. It should be noted that because some studies were published online ahead of time and the study publication date was a year or two behind, for statistical purposes, the year of publication of such research studies was included as the year of online publication. (e.g., a study shown in the reference as published in 2022 may have been published online in 2020).

## 3 Results

### 3.1 Number and type of publications

**FIGURE 2:**
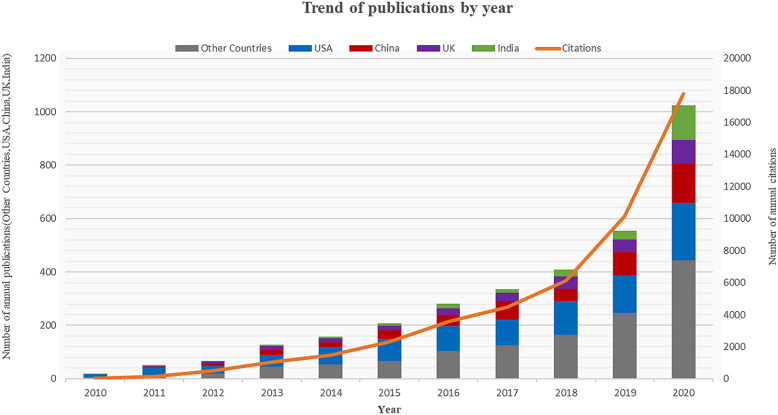
Annual trends in the number of articles published and citations related to drug repositioning.

Of the 2,978 papers obtained using the search criteria mentioned previously, the main ones were research studies (2248; 75.49%) and reviews (730; 24.51%). Furthermore, individual publications are not only classified by journals in the single category of research studies or reviews but also belong to other categories. These publications were also related to proceeding studies (68; 2.28%), early access (24; 0.81%), book chapters (7; 0.24%), data studies (2; 0.07%), and retracted publications (2; 0.07%). The vast majority of research studies and reviews were published in English (2967; 99.631%), with the remainder in Japanese (3; 0.101%), Chinese (2; 0.067%), Czech (1; 0.034%), French (1; 0.034%), German (1; 0.034%), Hungarian (1; 0.034%), Korean (1; 0.034%), and Portuguese (1; 0.034%). Ninety were from SSCI, and the remaining 2888 were from SCI-E. Further, 1,996 were from Open Access. An annual analysis of published research studies is shown in Figure 2. The number of publications for every year expanded from 17 in 2010 to 970 in 2020. Annual publications on the subject have increased by more than 64 times. The number of annual publications has been increasing at a relatively high rate since 2015, while in 2020, there was a spike in the number of publications and annual citations, probably due to the COVID-19 pandemic, a global public health emergency that prompted special attention from scientists. Among the four countries with the highest number of publications (the United States, China, the United Kingdom, and India), the United States has maintained a high growth volume since 2010, while China was the fastest in terms of average annual growth rate in the last three years. In 2020, the number of publications in India surged and surpassed the production of the United Kingdom.

### 3.2 Countries and number of publications

With respect to the 2978 publications related to drug repositioning research, 89 countries contributed to the field of drug repositioning research. The number of publications and citations from the 20 most productive countries/regions is shown in Table 1. There are nine countries/regions in Europe, five in the Americas, five in Asia, and one in Oceania.

**TABLE 1:** Top 20 most productive countries/regions in the field of drug repositioning.

Rank	Country	TP	TC	h-index	ACPP	nCC	SMCP (%)	Region
1	The United States	918	27,355	74	29.8	59	48.15	Anglo-America
2	P.R. China	485	11,147	49	22.98	39	36.70	Asia
3	The United Kingdom	284	8,762	43	30.85	57	69.01	Europe
4	India	247	3,203	27	12.97	37	30.77	Asia
5	Italy	232	6,024	39	25.97	40	47.41	Europe
6	Germany	171	5,213	36	30.49	50	67.25	Europe
7	South Korea	161	2221	21	13.8	24	29.20	Asia
8	Japan	146	3,037	26	20.8	22	25.34	Asia
9	Brazil	125	1911	24	15.29	29	42.20	Latin America
10	France	116	3,627	26	31.27	35	56.03	Europe
11	Canada	111	4,641	28	41.81	46	62.16	Anglo-America
12	Spain	109	2305	27	21.15	38	58.72	Europe
13	Australia	73	1816	23	24.88	36	79.45	Oceania
14	The Netherlands	73	1,559	22	21.36	37	75.34	Europe
15	Switzerland	59	2126	23	36.03	32	67.80	Europe
16	Sweden	58	1,434	19	24.72	37	86.21	Europe
17	Taiwan Region	58	1,110	17	19.14	8	36.21	Asia
18	Argentina	51	749	17	14.69	16	43.14	Latin America
19	Belgium	48	1,062	18	22.13	26	81.25	Europe
20	Mexico	47	1,162	19	24.85	15	42.55	Latin America

Notes: TP, total papers; TC, total citations; ACPP, average citations per publication; nCC, number of cooperative countries; and SMCP, share of multinational cooperation publications.

The four most productive countries/regions are, in order, the United States, China, the United Kingdom, and India. The United States is the absolute leader in this field, with 918 research studies on drug repositioning published since 2010, which is already more than the next highest number of publications in China and the United Kingdom combined. This is followed by India (247), Italy (232), Germany (171), South Korea (161), and Japan (146). Other productive countries include Brazil (125), France (116), Canada (111), Spain (109), Australia (73), the Netherlands (73), and Switzerland (59). In terms of publication impact, the United States led the Total citations (TC) rankings with 27,355, twice as many as that of China (11,147), which ranked second. We also included the average citations per publication (ACPP) in the comparison, which is calculated by dividing the TC by the TP (total papers) value and is a relative number that may better reflect the individual or collective level of attention than the individual TC and TP values. Canada ranked first in ACPP at 41.81, closely followed by the United Kingdom (30.85) and Germany (30.49). In addition, the h-index was originally proposed as a simple quantification that a researcher had at least h publications cited h times, reflecting to a certain extent the research results of the researcher as an individual (Hirsch, 2005). Later, the word “researcher” in the definition began to be replaced by collective words such as “academic group or institution (Van Raan, 2006)," “journal (Braun et al., 2006)," and “country (Csajbók et al., 2007)," becoming an indicator of the level of collective research to some extent. Undoubtedly, the h-index of the United States ranks first in this field with 74 times. Taking all parameters into account, we find that publications in the United Kingdom, the United States, and Canada perform better on average. While the number of publications in China and India is significant, they have received low levels of attention.

### 3.3 National/regional cooperation

It should be noted that DDA analysis software is nationally identified based on the location of each researcher’s institution address provided in the publication. If a publication is coauthored by institutions from more than two countries, the publication is defined as the result of an international collaboration. Whether there is some affiliation between the various institutions of the research group that produces the multicountry collaboration is not taken into account. As shown in Table 1, among the publications of the top 20 countries and regions, the proportion of international collaborations is quite high in European countries, especially in Sweden (86.21%) and Belgium (81.25%). Asian and Latin American countries are generally underrepresented. In addition, the United States, the most active country in publishing and the country with the most collaborations—with 59 countries or regions—still has over 50% of the studies published overall.

**FIGURE 3:**
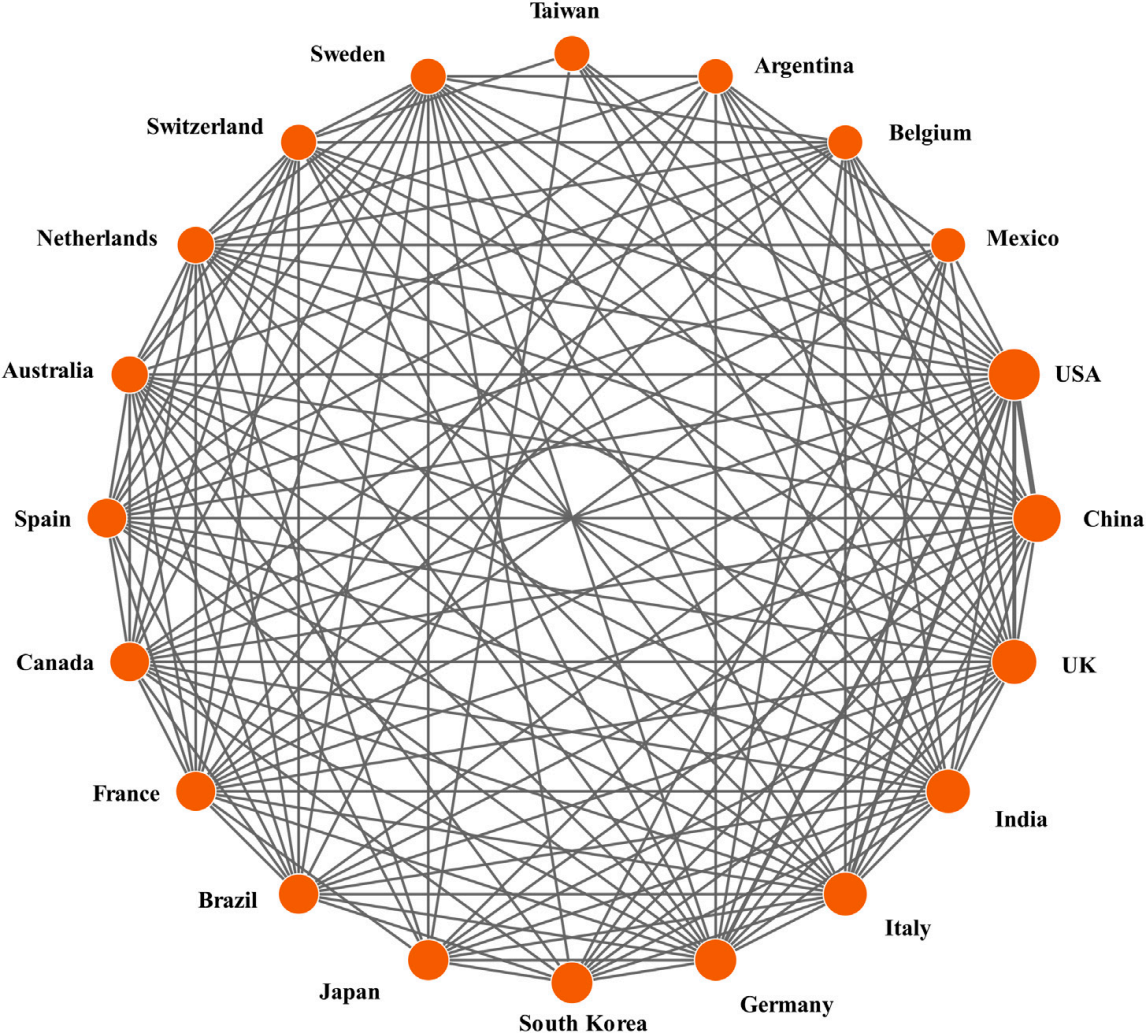
Cooperation between the top 20 most efficient countries/regions.

Figure 3 depicts the academic collaboration network for the top 20 countries and regions in terms of productivity. Using DDA software, the network was mapped using a co-occurrence matrix. The size of the circles is proportional to the extent of each country’s contribution, the lines between the circles represent the collaboration between countries/regions, and the thickness of the connecting lines indicates the frequency of collaboration (Bao et al., 2018). The results show that the United States cooperates most frequently with China and the United Kingdom and has the closest cooperation with them. In addition, Mexico, Belgium, Argentina, Taiwan, Japan, and Korea have slightly sparser cooperation networks among the 20 most productive countries/regions, while the remaining countries have more extensive cooperation networks among themselves.

### 3.4 Contributions of leading bodies

A total of 3,530 institutes were involved in drug repositioning research. The top 20 productive institutes are shown in Table 2. Eight of the top 20 institutions are from the United States, again indicating the dominance of the United States in drug repositioning research; three are from the United Kingdom; two are from China; and Brazil, France, Mexico, Canada, the Netherlands, Austria, and Sweden each have one. The Chinese Academy of Science ranked first in terms of the number of research studies, followed by Case Western Reserve University and the NIH. The Icahn School of Medicine at Mt Sinai ranked first in ACPP at 77.32. The NIH had the highest h-index value of 22. The Icahn School of Medicine at Mt Sinai was the best performer in ACPP at 77.32, followed by the University of California, San Francisco (67.82) and Johns Hopkins University (65.68), both of which are US-based research institutions. Compared with US research institutions, Chinese research institutions are at the back of the pack in terms of ACPP, and their impact needs to be improved.

**TABLE 2:** Top 20 most productive institutions in the field of drug repositioning for the period of 2010–2020.

Rank	Institution	TP	TC	ACPP	h-Index	PMCP (%)	Country/region
1	Chinese Acad Sci	54	1,286	23.81	19	98.15	China/Asia
2	Case Western Reserve Univ	38	1799	47.34	20	86.84	The United States/Anglo-America
3	NIH	37	1777	48.03	22	72.97	The United States/Anglo-America
4	Stanford Univ	35	1,401	40.03	16	80.00	The United States/Anglo-America
5	Univ Sao Paulo	34	452	13.29	13	76.47	Brazil/Latin America
6	Harvard Med Sch	33	1,078	32.67	18	84.85	The United States/Anglo-America
7	Univ Cambridge	32	788	24.63	14	90.63	The United Kingdom/Europe
8	Icahn Sch Med Mt Sinai	28	2165	77.32	15	75.00	The United States/Anglo-America
9	Kings Coll London	28	605	21.61	13	96.43	The United Kingdom/Europe
10	Aix Marseille Univ	27	1,183	43.81	15	92.59	France/Europe
11	Univ Nacl Autonoma Mexico	27	943	34.93	17	88.89	Mexico/Latin America
12	Shanghai Jiao Tong Univ	25	524	20.96	13	76.00	China/Asia
13	Univ Toronto	24	457	19.04	11	95.83	Canada/Anglo-America
14	Karolinska Inst	23	708	30.78	10	100.00	Sweden/Europe
15	Leiden Univ	23	327	14.22	11	78.26	The Netherlands/Europe
16	UCL	23	584	25.39	14	95.65	The United Kingdom/Europe
17	HM Pharma Consultancy	22	23	1.05	2	4.55	Austria/Europe
18	Johns Hopkins Univ	22	1,445	65.68	16	95.45	The United States/Anglo-America
19	NCI	22	602	27.36	14	100.00	The United States/Anglo-America
20	Univ Calif San Francisco	22	1,492	67.82	13	86.36	The United States/Anglo-America

Notes: TP, total papers; TC, total citations; ACPP, average citations per publication; and PMCP, Proportion of multi-institutional collaborative publications.

**FIGURE 4:**
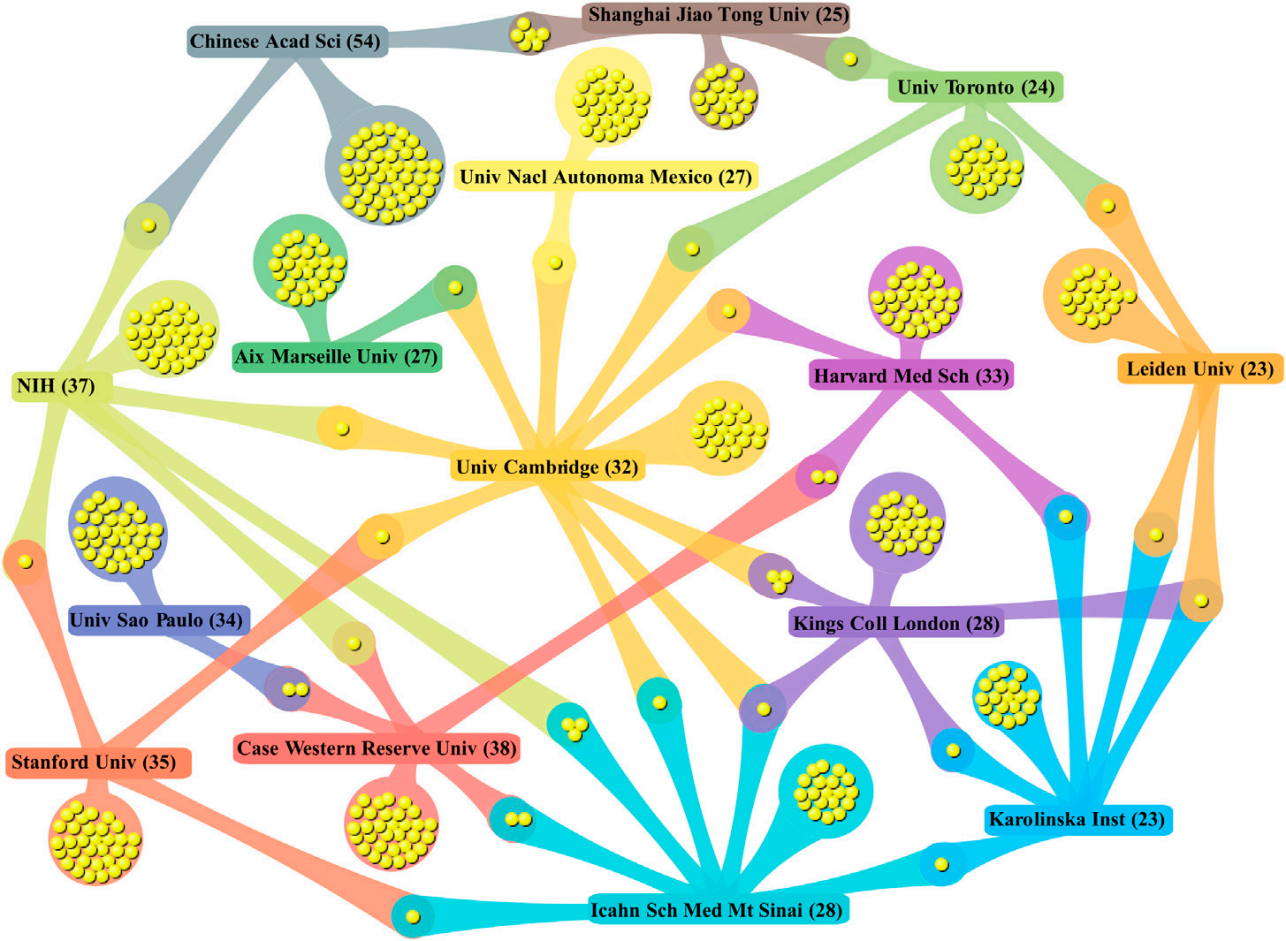
Collaboration matrix mapped between the first 15 productive bodies.

The collaboration network between the 15 largest institutions in 2010–2020 is shown in Figure 4. The collaboration network provides a more visual view of the collaboration with different institutions and thus helps in the search for more beneficial collaborations. Next to the name of each institution is its total number of publications. At the intersections of these institutions, yellow dots indicate collaborations with the other top 10 research institutions. It should be noted that the number of yellow dots can indicate the output of cooperation and the strength of interagency cooperation. The nodal data with no crossover points represent the number of publications produced by the institute, either by its independent work or in collaboration with research institutions outside the top 15 (Bao et al., 2019). From Figure 4, we see that the University of Cambridge established the largest collaborative network, followed by the large network established by four institutions, the NIH, the Icahn School of Medicine at Mt Sinai, Karolinska Institute, and King’s College London. In terms of the number of copublications with established institutions, the Chinese Academy of Science and Shanghai Jiao Tong University copublished as many as six, followed by the University of Cambridge and King’s College London and the NIH and the Icahn School of Medicine at Mt Sinai. Analyzing the aforementioned three pairs of institutional combinations, King’s College London has two publications that are the product of collaboration between the three research institutions. The University of Sao Paulo and Aix-Marseille University are relatively independent in this research area. Combining the ranking of multiple parameters, we found that the NIH and Icahn Sch Med Mt Sinai in the United States are the most vocal institutions in terms of academic research result perspective on the topic.

### 3.5 Contribution of leading research areas

An analysis of research areas gives a good indication of the scope of application of the research topic, with an unrestrained number of 74 areas covered, with the top 20 areas of research in terms of publication volume shown in Table 3. Briefly, “pharmacology and pharmacy” took first place with 962 articles, followed by “biochemistry and molecular biology”, and for ACPP, the top three were science and technology-other topics (36.1)", “mathematics (32.79)", and “cell biology (29.65)".

**TABLE 3:** Contribution of the top 20 research areas in the field of drug repositioning.

Rank	Research Area	TP	TC	ACPP	h-Index	SP%
1	Pharmacology & Pharmacy	962	25,243	26.24	67	32.3
2	Biochemistry & Molecular Biology	721	18,768	26.03	59	24.21
3	Oncology	302	7,104	23.52	40	10.14
4	Chemistry	274	5,539	20.22	33	9.2
5	Mathematical & Computational Biology	242	6,671	27.57	40	8.13
6	Science & Technology-Other Topics	234	8,448	36.1	42	7.86
7	Computer science	215	5,392	25.08	38	7.22
8	Biotechnology & Applied Microbiology	189	5,384	28.49	36	6.35
9	Cell biology	185	5,486	29.65	34	6.21
10	Research & Experimental Medicine	157	4,322	27.53	31	5.27
11	Microbiology	151	3,714	24.6	32	5.07
12	Neurosciences & Neurology	136	2513	18.48	26	4.57
13	Biophysics	114	2071	18.17	25	3.83
14	Genetics & Heredity	94	1878	19.98	23	3.16
15	Infectious diseases	93	2603	27.99	28	3.12
16	Immunology	68	1744	25.65	22	2.28
17	Mathematics	66	2164	32.79	27	2.22
18	General & Internal Medicine	64	1,299	20.3	21	2.15
19	Parasitology	56	1,079	19.27	18	1.88
20	Virology	56	1,079	19.27	18	1.88

Notes: TP, total papers; TC, total citations; ACPP, average citations per publication; and SP%, share of publications.

**FIGURE 5:**
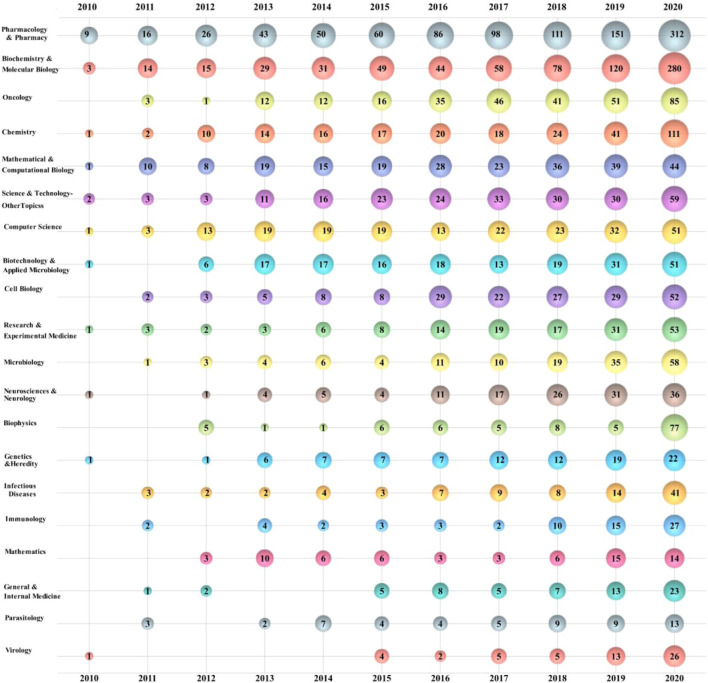
Bubble chart of the top 20 drug repositioning research areas by year.

Figure 5 shows a bubble graph of the top 20 drug repositioning research areas. The bubble plot shows three dimensions of the data, namely, research area, year of publication, and the number of publications. The horizontal change in bubble size illustrates the growing trend of research areas over time, the vertical size of the bubble shows the most popular research areas in that year, and the number in the bubble indicates the frequency of the topic in the research area and the number of publications in that year (Chen et al., 2016). The number of research results in each relevant field is increasing year by year. Biophysics increased from five in 2019 to 77 in 2020, a more than 15-fold increase, suggesting that drug repositioning may have made a breakthrough or become widely used in this field. The field of virology was in a downturn from 2010 to 2014, with only one publication, with a gradual increase in relevant studies after 2015.

### 3.6 Contribution of major journals

**FIGURE 6:**
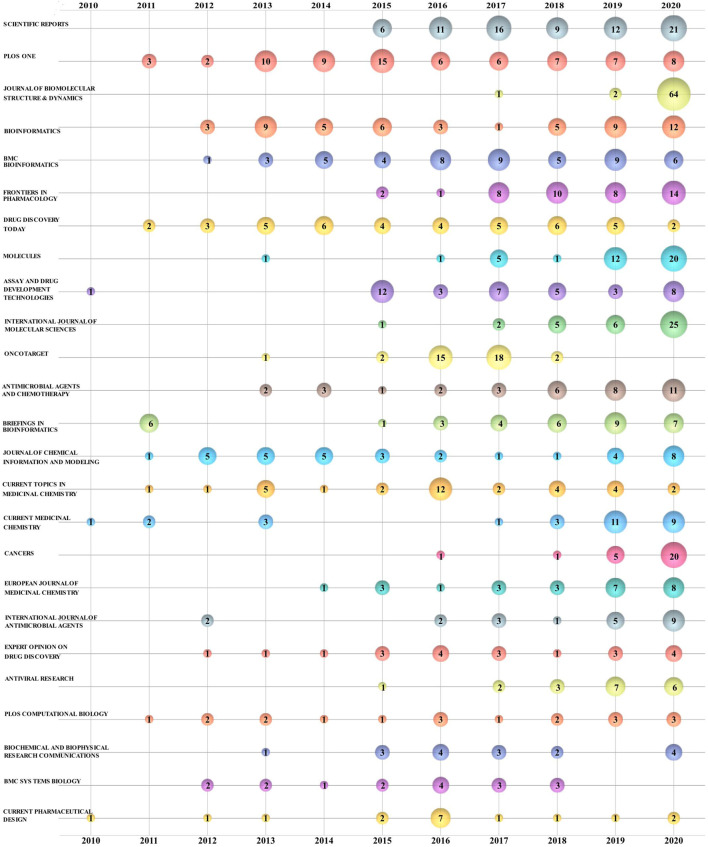
Bubble chart of the top 25 drugs repositioned by year in terms of journal production.

For scholars studying drug repositioning-related topics, knowing which journals publish relevant research is important in deciding which journals to read or submit their research studies to. A total of 2,988 publications related to drug repositioning research were published in 845 journals during the period of 2010–2020. The top 25 journals in terms of a total number of studies published are shown in Table 4 Sci Rep topped the list with 75 studies published, followed by *PLoS One* (73; 2.52%), *J. Biomol. Struct. Dyn* (67; 2.45%), *Bioinformatics* (53; 2.25%), and *BMC bioinformatics* (50 articles; 1.78%). The rest of the journals had a share of less than 1.5%. In terms of total citations (TC), at present, studies in *Drug Discov.* have been cited a total of 2,119 times over the past 10 years, followed in rank by PLoS One (1800) and Bioinformatics (1,677). For ACPP, *Drug Discov. Today* still holds first place with a high frequency of 50.45 times, followed by *PLoS Comput* (33.14 times). The impact factor (IF) of a journal is calculated by dividing the total number of citations of all publications in the journal in the previous two years by the number of publications (Garfield, 2006). Thus, Table 4 shows that the ACPP of drug repurposing publications included in most journals is much higher than that of IF, which roughly verifies that the number of scholars interested in drug repurposing is relatively high. In terms of the impact factor (IF) of specific journals, except for *Oncotarget* and B*MC Syst. Biol.*, which have not been included in SCI since 2018 and 2020, *Brief. Bioinform.* has the highest value of 11.622, followed by *Drug Discov. Today* (7.851), *Bioinformatics* (6.937), *Cancers* (6.639), *Eur. J. Med. Chem* (6.514), and *Expert. Opin. Drug Discov.* (6.098). The bubble chart shows that *J. Biomol. Struct. Dyn.* featured 64 publications in 2020, compared to a combined total of only four publications in the previous ten years; the Oncotarget journal inclusion in this category peaked in 2016–2017 (Figure 6).

**TABLE 4:** Top 25 journals publishing studies in drug repositioning studies.

Rank	*Journal Title*	TP	TC	ACPP	IF (2020)
1	*Sci Rep*	75	1,081	14.41	4.38
2	*PLoS One*	73	1800	24.66	3.24
3	*J. Biomol. Struct. Dyn*	67	1,000	14.93	3.110
4	*Bioinformatics*	53	1,677	31.64	6.937
5	*BMC Bioinformatics*	50	658	13.16	3.169
6	*Front. Pharmacol*	43	1,073	24.95	5.811
7	*Drug Discov. Today*	42	2119	50.45	7.851
8	*Molecules*	40	329	8.23	4.412
9	*ASSAY DRUG DEV. TECHNOL.*	39	224	5.74	1.738
10	*Int. J. Mol. Sci.*	39	785	20.13	5.924
11	*Oncotarget*	38	861	22.66	—
12	*Antimicrob. Agents Chemother*	36	770	21.39	5.191
13	*Brief. Bioinform*	36	1,585	44.03	11.622
14	*J. Chem Inf. Model.*	35	1,134	32.4	4.956
15	*Curr. Top. Med. Chem.*	34	447	13.15	3.295
16	*Curr. Med. Chem.*	30	343	11.43	4.53
17	*Cancers*	27	185	6.85	6.639
18	*Eur. J. Med. Chem.*	26	418	16.08	6.514
19	*Int. J. Antimicrob. Agents*	22	729	33.14	5.283
20	*Expert. Opin. Drug Discov.*	21	366	17	6.098
21	*Antiviral Res.*	19	312	16.42	5.927
22	*PLoS Comput. Biol.*	19	889	46.79	4.475
23	*Biochem. Biophys. Res. Commun.*	17	218	12.82	3.575
24	*BMC Syst. Biol.*	17	407	23.94	—
25	*Curr. Pharm. Design*	17	312	18.35	3.116

Notes: TP, total papers; TC, total citations; ACPP, average citations per publication; and IF: impact factor.

### 3.7 Contribution of the lead author

For scholars interested in the topic of drug repositioning, it is useful to know how other researchers are working on the issue to facilitate communication and collaboration between scholars. A total of 15,620 authors contributed to studies within our measurement consideration, and Table 5 shows the top 20 prolific authors by a number of publications. Of these 20 highly productive authors, seven were from the United States, three were from Argentina, and two were from Germany, indicating a relatively high concentration of drug repositioning research in certain countries. In addition, the NIH (United States), Case Western Reserve University (United States), Tech University Dresden (Germany), and the National University of La Plata (Argentina) each have two of these academics.

**TABLE 5:** Contribution of the top 20 authors to drug repurposing studies.

Rank	Author	TP	TC	ACPP	H-Index	TPR	Institution (Current), Country/Region
1	Cheng, FX	25	2514	100.56	21	17	Case Western Reserve Univ, USA/Anglo-America
2	Talevi, A	23	446	19.39	12	17	Natl Univ La Plata UNLP, Argentina/Latin America
3	Mucke, HAM	22	23	1.05	2	22	HM Pharma Consultancy, Austria/Oceania
4	Zheng, W	19	1,189	62.58	17	12	NIH,USA/Anglo-America
5	Xu, R	16	330	20.63	11	15	Case Western Reserve Univ, USA/Anglo-America
6	Dudley, JT	15	1,218	81.2	10	7	Icahn Sch Med Mt Sinai, USA/Anglo-America
7	Schroeder, M	15	454	30.27	11	12	Tech Univ Dresden, Germany/Europe
8	Andre, N	12	471	39.25	9	5	Aix Marseille Univ, France/Europe
9	Wang, QuanQiu	12	237	19.75	9	0	ThinTek LLC,USA/Anglo-America
10	Arga, KY	11	175	15.91	8	6	Marmara Univ, Turkey/Asia
11	Haupt, V. Joachim	11	399	36.27	8	0	Tech Univ Dresden, Germany/Europe
12	Carrillo, C	10	192	19.2	8	1	Inst Ciencias and Tecnol Cesar Milstein, Argentina/Latin America
13	Duenas-Gonzalez, A	10	326	32.6	8	9	Univ Nacl Autonoma Mexico, Mexico/Latin America
14	Bellera, Carolina L	10	192	19.2	7	0	Natl Univ La Plata, Argentina/Latin America
15	Sun, Wei	10	508	50.8	8	0	NIH,USA/Anglo-America
16	Tang, Y	10	950	95	9	6	East China Univ Sci and Technol, Peoples R China/Asia
17	Tempone, AG	10	113	11.3	7	7	Adolfo Lutz Inst, Ctr Parasitol and Mycol, Brazil/Latin America
18	Aittokallio, T	9	431	47.89	8	6	Aalto Univ, Finland/Europe
19	Bae, JS	9	39	4.33	4	9	Kyungpook Natl Univ, South Korea/Asia
20	Butte, AJ	9	1,389	154.33	9	5	Univ Calif San Francisco, USA/Anglo-America

Notes: TP, total papers; TC, total citations; ACPP, average citations per publication; and TPR, total number of publications for which they are responsible.

Cheng, FX leads the list with 25 research studies, followed by Talevi, A (23) and Mucke, HAM (22). For the list of corresponding authors, the top three remain, in order, Mucke, HAM (22), Cheng, FX (17), and Talevi, A (17). In terms of ACPP ranking, Butte, AJ was ranked first with 154.33 points, followed by Cheng, FX (100.56), Tang, Y (95), and Dudley, JT (82). Cheng, FX still has the highest h-index at 21, followed by Zheng, W (17), Talevi, A (12), Xu, R (11), and Schroeder, M (11). The h-index has two drawbacks when researchers of the same topic are compared with each other (Bornmann and Daniel, 2007). One is that the scholar’s h-index does not decrease over time but only grows or stays the same, and it is not possible to obtain information on whether the scholar is still in an academic career. In this study, we narrow the study to the most recent publications from 2010 to 2020, taking into account the timeliness of the h-index response information. Second, older scholars usually enter academia earlier and have an advantage in their h-indexes in comparison with those of younger scholars. Therefore, this phenomenon must be targeted for analysis or illustration. Thus, by combining the authors’ educational experiences and employment relationship changes that were recorded in the WOS database and ORCID business cards, we inferred that more than half of the scholars in the top 20 in terms of the number of publications received their Ph.D. before 2008, and two scholars, Mucke, HAM and Zheng, Wei, are older. In contrast, Cheng, FX, a scholar from Case Western Reserve Univ, completed his Ph.D. without a gap in 2013 and may have a longer academic career in the future; therefore, Cheng, FX’s h-index in the field of drug repositioning is likely to grow more in the future and Cheng, FX is likely to have more academic influence.

### 3.8 Research hotspots and trends

To reveal the focus of drug repositioning research and research trends, the author keywords and the highly cited and hot research topics of the ESI for each of the 2978 publications were analyzed, which were also derived from the core database of the WOS database (SCI-E/SSCI) (Liao et al., 2019). Highly cited studies were defined as studies in the top 1% of the citations for all studies in the same ESI discipline within the 10-year range of inclusion of ESI inclusion (Chang et al., 2020). A hot research topic of the ESI refers to a study published in two years with a citation frequency within one of the corresponding disciplines in the world in the last two months (Li L. et al., 2020).

#### 3.8.1 Author keyword analysis

**FIGURE 7:**
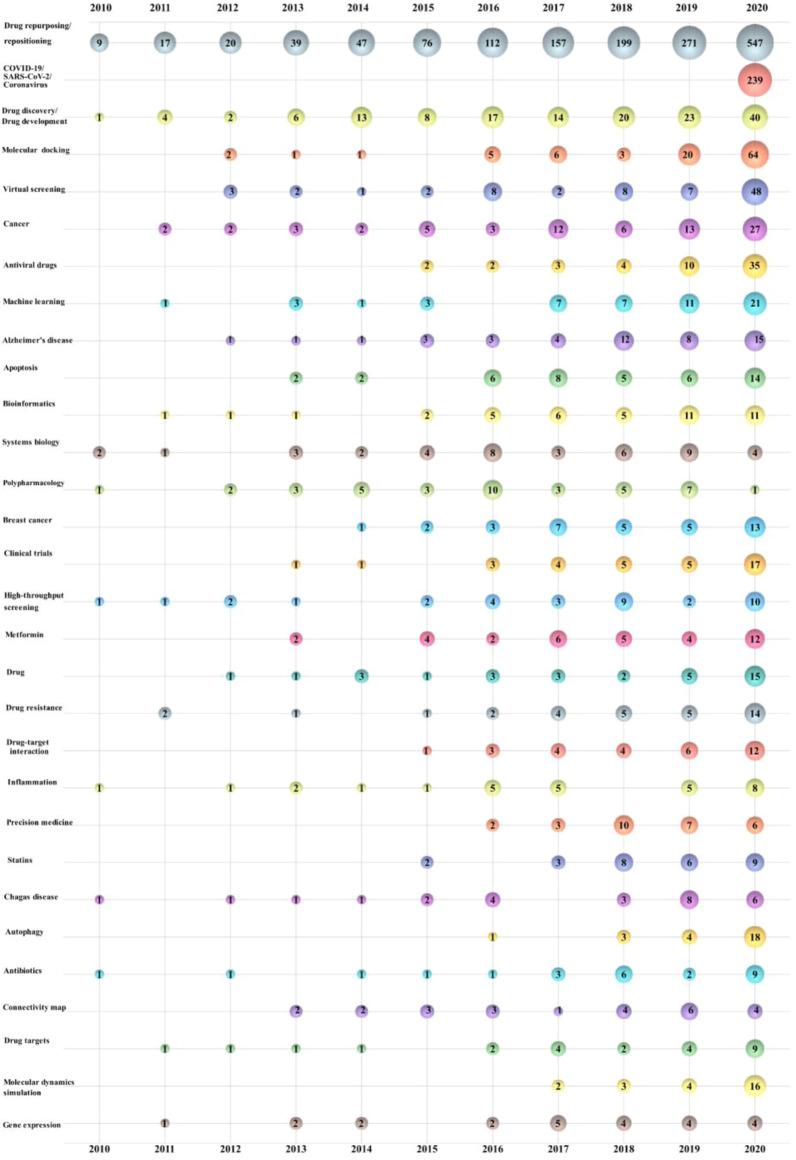
Bubble chart of the top 30 author keywords by year.

Author keywords tend to provide more information and have thus become a widespread focus (Chen et al., 2021; Zhen et al., 2022). The data of 6,083 author keywords in the search results were merged to make keywords with the same meaning represented by a single unified word. In the end, 5,616 author keywords were obtained. It should be specified that some publications without author keywords were excluded from the statistical analysis. Of these author keywords, 4,296 were used only once, representing 76.50% of the total. A total of 1,216 (21.65%) appeared 2–10 times, 79 (1.41%) appeared 10–20 times, 37 (0.66%) appeared 21–50 times, and the remaining eight (0.14%) were used between 51 and 1,500 times. All keywords cumulatively appear a total of 12,400 times, while the top 30 most used author keywords appear 2,967 times alone, or approximately 23.93%, as shown in Figure 7. The comparison of keywords in recent years allows for tracking the frontiers of research and predicting hotspots and trends in drug repositioning research. The bubble plots show the three dimensions of the data, namely, the year of publication, the author’s keywords, and the number of corresponding publications. The horizontal change in the size of the bubble illustrates the increasing trend of author keywords over time, the vertical size of the bubble shows the most popular keywords in that year, and the numbers in the bubble indicate the frequency of author keywords and the number of publications.

The top 30 keywords include five diseases: “COVID-19/SARS-CoV-2/Coronavirus” (239), “Cancer” (75), “Alzheimer’s disease” (48), “Breast cancer (36)", and “Chagas disease” (27). Drug names appear four times, “Antiviral drugs” (56), “Metformin” (35), “Statins” (28), and “Antibiotics” (25), which reveal the diseases and applications to which drugs were often repositioned during these 11 years. There were four subject categories, “Bioinformatics” (43), “Polypharmacology” (42), “Systems biology” (42), and “Precision medicine” (28) and eight occurrences of research methods, namely, “Virtual screening” (81), “Molecular docking” (64), “Machine learning” (54, eighth), “Clinical trials” (36), “High-throughput screening” (35), “Connectivity map” (28), and “Molecular dynamics simulation” (26).

In the context of the pandemic in 2020, there was a surge in research on the subject, with “COVID-19/SARS-CoV-2/Coronavirus” topping the list of keywords as soon as they appeared that year. “Virtual screening” is a research method that appeared seven times more frequently in 2020 than in the previous year. Since “Drug repurposing/repositioning” is a subject matter and a strategy for drug discovery/drug development, it would not make much sense to analyze these two keywords. Molecular docking is one of the core steps of virtual screening, and the COVID-19 pandemic generated many opportunities for the practice of drug repositioning. Therefore, high-quality studies of the keywords “COVID-19/SARS-CoV-2/Coronavirus”, “Virtual screening”, and “Molecular docking” were surveyed, as shown in the bubble chart, in the past two years, reflecting the relevant research trend in recent years. Wang, F et al. developed a new free reverse docking server based on a consensus algorithm (combining several docking algorithm strategies) to address the original shortcomings of computational molecular docking in drug repositioning, such as a low success rate, cumbersome operational steps, and reliance on code writing (Wang et al., 2019). M Lapillo et al. extensively evaluated the performance assessment of docking-based target fishing methods and developed a consensus docking-based target fishing tactic (Lapillo et al., 2019). In a virtual screening process, Gervasoni, S. conducted a literature search for molecular binding sites for SARS-CoV-2-associated protein targets while combining pocket and docking searches to propose a new pocket mapping strategy that identifies binding cavities with significantly better performance than pocket detection alone (Gervasoni et al., 2020). Xie, L et al. screened antitoxic drugs based on the multitarget structure of the pathway center and stated that this inhibition of multiple targets in one pathway would be more effective than targeting a single protein, and the chance of drug resistance was smaller, which could be applied to other pathways (Xie and Xie, 2019). Li, Z et al. reported a virtual screening method based on accelerated free energy perturbation absolute binding free energy (FEP-ABFE) prediction and stated that the virtual screening method based on the prediction of FEP-ABFE will play a role in many other drug repositioning studies (Li Z. et al., 2020). After a series of drug repurposing computational screens and various validation activities by several scientists, it was agreed that raltegravir (Beck et al., 2020; Elfiky, 2020), clonidine (Jeon et al., 2020; Xu et al., 2020), chloroquine and hydroxychloroquine (Fantini et al., 2020) have therapeutic effects in the treatment of novel coronavirus.

In addition, from the studies on the keyword “Machine learning” over the 10-year period shown in the bubble chart, it was found that the classical machine learning algorithms of support vector machines (Kinnings et al., 2011; Pérez-Sánchez et al., 2014; Zhao and So, 2018), regularized least squares (Hao et al., 2016; Zhou et al., 2019), logistic regression (Qabaja et al., 2014; Liu et al., 2015; Xu et al., 2017), and random forests (Cao et al., 2014; Coelho et al., 2016) have been widely used in inferring drug–target and drug–disease interactions.

#### 3.8.2 Analysis of hot research topics

While the level of influence of a study is reflected by a combination of many aspects, the number of citations remains an important indicator (Wu Y. et al., 2020). Based on the definition of highly cited and hot ESI papers in Section 3.8 of this study, a total of 108 highly cited studies were obtained, of which 11 were hot research topics. Hot research topics are shown in Table 6. It should be noted that the first-ranked author is used here as a representative, and the corresponding institution is shown. This rule is followed in Section 3.8.3 of this study. All hot research topics were published in 2020, and with the exception of an article describing the damage caused by nonsteroidal anti-inflammatory drugs (NSAIDs) to multiple organs and new information on drug repurposing (Bindu et al., 2020), the remaining studies focused on drug repositioning therapeutic target studies in novel coronavirus pneumonia (Wu C. et al., 2020; Gordon et al., 2020), screening drug studies (Elfiky, 2020; Jeon et al., 2020; Rut et al., 2020; Singh et al., 2020; Wang, 2020), reviews of clinical trials (Rosa and Santos, 2020; Tu et al., 2020), and reports of other coronavirus therapeutic agents and vaccine studies (Liu et al., 2020). From the perspective of cooperation, most of them were completed by a country’s independent agency. In terms of the countries and regions studied, four studies involved US scholars, five studies involved Asian scholars, and one contribution was from an African scholar. In addition, “A SARS-CoV-2 protein interaction map reveals targets for drug repurposing (Gordon et al., 2020)”, published in *Nature* by Gordon, DE with a total of 125 scholars from the United States, the United Kingdom, and France was the most cited publication with 952 citations.

**TABLE 6:** All ESI hot citation studies from 2011 to 2020.

No	Author	Title	TC	Journal	Institution,Country/Region	OPC
1	Gordon, DE et al.	A SARS-CoV-2 protein interaction map reveals targets for drug repurposing	952	*Nature*	Univ Calif San Francisco, United States et al.	France; England
2	Wu, CR et al.	Analysis of therapeutic targets for SARS-CoV-2 and discovery of potential drugs by computational methods	817	*Acta Pharm. Sin. B*	Huazhong Univ Sci and Technol, Peoples R China et al.	None
3	Liu, C et al.	Research and Development on Therapeutic Agents and Vaccines for COVID-19 and Related Human Coronavirus Diseases	543	*ACS Central Sci*	CAS, United States	None
4	Elfiky, AA	Ribavirin, Remdesivir, Sofosbuvir, Galidesivir, and Tenofovir against SARS-CoV-2 RNA dependent RNA polymerase (RdRp): A molecular docking study	363	*Life Sci*	Cairo Univ, Egypt	None
5	Tu, YF et al.	A Review of SARS-CoV-2 and the Ongoing Clinical Trials	324	*Int. J. Mol. Sci*	Natl Yang Ming Univ, Taiwan	None
6	Jeon, S et al.	Identification of Antiviral Drug Candidates against SARS-CoV-2 from FDA-Approved Drugs	211	*Antimicrob. Agents Chemother*	Inst Pasteur Korea, South Korea	None
7	Wang, JM	Fast Identification of Possible Drug Treatment of Coronavirus Disease-19 (COVID-19) Through Computational Drug Repurposing Study	199	*J. Chem. Inf. Model*	Univ Pittsburgh, United States	None
8	Rosa, SGV et al.	Clinical trials on drug repositioning for COVID-19 treatment	131	*Rev. Panam. Salud Publica*	Univ Fed Fluminense, Brazil	None
9	Singh, TU et al.	Drug repurposing approach to fight COVID-19	86	*Pharmacol. Rep*	ICAR Indian Vet Res Inst, India	None
10	Rut, W et al.	Activity profiling and crystal structures of inhibitor-bound SARS-CoV-2 papain-like protease: A framework for anti-COVID-19 drug design	69	*Sci. Adv*	Wroclaw Univ Sci and Technol, Poland et al.	The United States
11	Bindu, S et al.	Non-steroidal anti-inflammatory drugs (NSAIDs) and organ damage: A current perspective	63	*Biochem. Pharmacol*	Bose Inst, India et al.	None

Notes: TC, total citations; and OPC, other partner countries.

#### 3.8.3 Analysis of the most cited studies

Eleven hot research topics were removed from the 108 highly cited ESI studies, and the top 20 most cited studies were selected from the remaining highly cited studies for analysis. In terms of year of publication, the study by Dudley, JT et al. published in *NUCLEIC ACIDS RESEARCH* in February 2011 was the earliest of these studies (Dudley et al., 2011). Five highly cited studies were published in 2013, and three studies were published as recently as 2020. Two studies were published in *Nature*, and one each was published in *Nat. Rev. Drug Discov.* and *Nat. Med. subj. of Nature E*; *J. Med. Chem. L* was next with two studies. There were 12 studies with the first author or coauthor from the United States, representing more than half of those in Table 7, followed by China (4), Canada (2), England (2), Germany (2), Japan (2), and Switzerland (2) in order of contribution of two or more studies. Nine studies were based on collaborations between different institutions in multiple countries. One of them, entitled “Alcohol-abuse drug disulfiram targets cancer via p97 segregase adapter NPL4”, published in *Nature* in 2017 by Skrott, Z et al. is a collaboration between scholars from six countries: Czech Republic, the United States, Denmark, Sweden, Switzerland, and China (Skrott et al., 2017). In TC, “DrugBank 5.0: a major update to the DrugBank database for 2018” (Wishart et al., 2018) by Canadian University of Alberta scientists Wishart, DS et al. ranked first (1820 total citations). The most cited publication on an annual basis was “Network-based drug repurposing for novel coronavirus 2019-nCoV/SARS-CoV-2”, published in 2020, which was authored by Zhou, YD et al. and was the highest annual average cited publication with 609 citations (Gordon et al., 2020). The scientists Cheng, FX and Dudley, JT, contributed to two of these 20 publications and are important influencers in the field.

**TABLE 7:** Top 20 highly cited ESI publications from 2011 to 2020.

No	Author (PY)	Title	TC	TCPY	Journal	Institution,Country/Region	OPC
1	Wishart, DS et al. (2018)	DrugBank 5.0: a major update to the DrugBank database for 2018	1820	606.7	*Nucleic Acids Res*	Univ Alberta, Canada et al.	None
2	Pushpakom, S et al. (2019)	Drug repurposing: progress, challenges and recommendations	885	442.5	*Nat. Rev. Drug Discov*	Univ Liverpool, England et al.	None
3	Maier, L et al. (2018)	Extensive impact of non-antibiotic drugs on human gut bacteria	639	213.0	*Nature*	European Mol Biol Lab, Germany et al.	Japan
4	Zhou, YD et al. (2020); Cheng, FX et al. (2020)	Network-based drug repurposing for novel coronavirus 2019-nCoV/SARS-CoV-2	609	609.0	*Cell Discov*	Cleveland Clin, United States et al.	None
5	Anighohro, A et al. (2014)	Polypharmacology: Challenges and Opportunities in Drug Discovery	492	70.3	*J. Med. Chem*	Univ Modena and Reggio Emilia, Italy et al.	Germany
6	Cheng, FX et al. (2012)	Prediction of Drug-Target Interactions and Drug Repositioning via Network-Based Inference	491	54.6	*PLoS Comput. Biol*	E China Univ Sci and Technol, Peoples R China	None
7	Langhans, SA (2018)	Three-Dimensional *in Vitro* Cell Culture Models in Drug Discovery and Drug Repositioning	395	131.7	*Front. Pharmacol*	Alfred I DuPont Hosp Children, United States	None
8	Xu, M et al. (2016)	Identification of small-molecule inhibitors of Zika virus infection and induced neural cell death via a drug repurposing screen	389	77.8	*Nat. Med*	NIH, United States et al.	China
9	Sirota, M et al. (2011); Dudley, JT et al. (2011)	Discovery and Preclinical Validation of Drug Indications Using Compendia of Public Gene Expression Data	327	32.7	*Sci. Transl. Med*	Stanford Univ, United States	None
10	Sriram, K et al. (2018)	G Protein-Coupled Receptors as Targets for Approved Drugs: How Many Targets and How Many Drugs?	311	103.7	*Mol. Pharmacol*	Univ Calif San Diego, United States	None
11	Dudley, JT et al. (2011)	Exploiting drug-disease relationships for computational drug repositioning	282	28.2	*Brief. Bioinform*	Arizona State Univ, United States et al.	None
12	Medina-Franco, JL et al. (2013)	Shifting from the single to the multitarget paradigm in drug discovery	285	35.6	*Drug Discov. Today*	Univ Nacl Autonoma Mexico, Mexico et al.	The United States
13	Peters, JU (2013)	Polypharmacology - Foe or Friend?	275	34.4	*J. Med. Chem*	F Hoffmann La Roche Ltd., Switzerland	None
14	Yoshida, GJ et al. (2015)	Metabolic reprogramming: the emerging concept and associated therapeutic strategies	255	42.5	*J. Exp. Clin. Cancer Res*	Japan Soc Promot Sci, Japan	None
15	Skrott, Z et al. (2017)	Alcohol-abuse drug disulfiram targets cancer via p97 segregase adaptor NPL4	249	62.3	*Nature*	Palacky Univ/Czech Republic et al.	Denmark; Sweden; Switzerland; The United States; China
16	Li, J et al. (2016)	A survey of current trends in computational drug repositioning	242	48.4	*Brief. Bioinform*	Chinese Acad Med Sci, Peoples R China et al.	The United States
17	Stokes, JM et al. (2020)	A Deep Learning Approach to Antibiotic Discovery	235	235	*Cell*	MIT, United States et al.	Canada
18	Reddy, AS et al. (2013)	Polypharmacology: drug discovery for the future	228	28.5	*Expert Rev. Clin. Pharmacol*	Univ Texas Houston, United States	None
19	Menden, MP et al. (2013)	Machine Learning Prediction of Cancer Cell Sensitivity to Drugs Based on Genomic and Chemical Properties	229	28.6	*PLoS One*	Wellcome Trust Genome Campus Cambridge, England et al.	The United States
20	Beck, BR et al. (2020)	Predicting commercially available antiviral drugs that may act on the novel coronavirus (SARS-CoV-2) through a drug-target interaction deep learning model	225	225	*Comp. Struct. Biotechnol. J*	Deargen Inc., South Korea et al.	The United States

Notes: PY, publication year; TC, total citations; TCPY, total citations per year; and OPC, other partner countries.

The three studies published in 2020 focus on novel coronavirus-related drug rediscovery activities (Zhou et al., 2020) and the use of deep learning techniques (Beck et al., 2020; Stokes et al., 2020). Dudley, JT et al. (2011) and Pushpakom, S et al. (2019) provided systematic reviews of the methods and challenges of drug repositioning at that time (Dudley et al., 2011; Pushpakom et al., 2019). Initially, Sirota, M et al. (2011) explored the role of integrating genome-wide computational approaches for predicting reusable drugs (Sirota et al., 2011), while from 2013 onward, Peters, JU et al., Medina-Franco et al., JL et al., Reddy, AS et al., and Anighoro, A et al. generally recognized the importance of combining multiple points of pharmacological knowledge for drug repositioning studies (Medina-Franco et al., 2013; Peters, 2013; Reddy and Zhang, 2013; Anighohro et al., 2014). In the face of a worldwide health emergency caused by the Zika virus epidemic, Xu et al. (2016) used drug repositioning to identify lead compounds for drug development (Xu et al., 2016). Of course, techniques related to the mining of repositionable drugs through experimental high-throughput screening, a traditional experimental approach, are not without progress; for example, Langhans (2018) explored the challenges of transferring 3D cell culture technology to the use of high-throughput screening (HTS) (Langhans, 2018).

## 4 Discussion

In 1995, Mchugh et al. investigated the immunomodulatory action mechanism of thalidomide in humans, which was the first relevant publication on drug repositioning (Mchugh et al., 1995). The publication time can be divided into three phases: the growth period of 1995–2009, the steady growth period of 2010–2018, and the rapid rise from 2019 and beyond. The 2978 publications studied between 2010 and 2020 were completed by 15,338 authors from 3,530 research institutions in 89 countries, and at the time of this study’s completion, the WOS database had surpassed more than 1,400 publications in 2021 under the same search restrictions for the topic, with more than 31,000 citations for the year, supporting further evidence that the topic is still gaining momentum worldwide.

The publication countries/regions are divided into three types: first, countries with a traditionally developed medical level, mainly developed countries in Western Europe, North America, and Oceania; second, countries with a developed pharmaceutical manufacturing industry, such as India and Japan in Asia; and third, developing countries with some research potential, such as China, Brazil, Argentina, and Mexico. In terms of national cooperation, Western European countries have shown a high degree of cooperation, with the United States, China, and the United Kingdom cooperating more frequently. This may be because Western European countries have a tradition of cooperation in the field of research, and the United States, China, and the United Kingdom are the most powerful countries in terms of drug repositioning publications and therefore cooperate more with each other. The United States accounts for half of the 20 most productive institutions, which may explain why the United States still publishes more than 50% of its studies independently, despite having the largest international collaborative network base, because it already has the most active and high-quality producing institutions within the country for research institutions seeking collaboration. Furthermore, 19 of these 20 institutions are universities and research institutes, and one is a company, HM Pharma Consultancy, which was established in 2000 to focus on drug repositioning for the development of new drugs (Nosengo, 2016). This evidence suggests that the topic of drug repositioning is not only widely studied in academia but also has a place in the industry.

The 2978 studies are spread across 74 research areas, but pharmacology and pharmacy and biochemistry and molecular biology account for a larger proportion of the total number of studies. It is quite notable that the majority of studies reported in biophysics did not rise significantly until 2020. The reasons for this may be the following: first, there was a breakthrough in basic research in this field in 2020 and second, due to the novel coronavirus, research in this direction has increased its application for the prevention and control of the pandemic.

In terms of journals, *Sci Rep* ranked first, followed by *PLoS One* and *J. Biomol. Struct. Dyn*. In terms of lead authors, three have the most productive and influential positions: Cheng, FX is the most prolific author, based on the number of papers and h-index; Mucke, HAM is the most frequent corresponding author; and Butte, AJ is the top author in terms of ACPP ranking. Even though Latin American countries do not have an advantage in terms of national cooperation or the total number of institutional funding units, Latin American scholars have overcome many obstacles and are actively at the forefront of scholarship, contributing significantly to the field.

Through the analysis of the authors’ keywords, cancer has been the main disease addressed by this method. Metformin has been found by many scientists to have a good inhibitory effect on various tumors, mainly in gynecology (Kumar et al., 2013; Xu et al., 2015; Gadducci et al., 2016; Seliger et al., 2019), and it has become a specific drug that has been most frequently mentioned in drug reuse in recent years. In terms of “antiviral drugs”, scholars not only use drug repositioning to find antiviral drugs to treat diseases, such as Ebola (Kouznetsova et al., 2014; Dyall et al., 2018) and HIV (Trivedi et al., 2020), that have plagued humans for a long time but also use this method to seek treatments for infectious diseases, such as Zika virus (Xu et al., 2016; Chan et al., 2017) and novel coronaviruses that have threatened several countries and even the world. For these diseases, emergency research on drug repositioning has played an important role in reducing mortality, calming patient fears, and restoring economic production when no specific drugs or vaccines were initially available during the pandemic. The combination of precision medicine and drug repositioning studies, often used to seek treatments for rare diseases (Álvarez-Machancoses et al., 2020) and, in particular, genetically related diseases (Reay et al., 2020), is expected to be fully developed in the future. In the past 2 years, “Virtual screening”, together with “Molecular docking” and “Machine learning”, has become the most cutting-edge and important research methods in related technology fields, constantly improving the accuracy of drug reuse and screening. Currently, to develop more efficient and accurate research, there are two trends in the use of drug repositioning. One is the combination of various methods, such as the use of text mining and network analysis, and the creation of statistical models for predicting semantic link association to assess the relationship between pharmacological target pairings (Chen et al., 2012); text analysis combined with machine learning (Zhu et al., 2020) to develop drugs for Parkinson’s disease; prediction of new DTIs using data from multiple databases (Olayan et al., 2018); and the obtained relocated anticancer drugs were verified by cross-validation, literature, and experimental verification (Cheng et al., 2021). Second, the most advanced algorithms are applied and improved, such as matrix decomposition (Xuan et al., 2019; Huang et al., 2020; Meng et al., 2021; Tang et al., 2021; Sadeghi et al., 2022) and matrix completion (Luo et al., 2018; Yan et al., 2022) and deep learning (Aliper et al., 2016; Zeng et al., 2019; Chiu et al., 2020; Stokes et al., 2020; Lee and Chen, 2021; Liu et al., 2021).

In fact, some of the studies in the list of highly cited research topics on novel coronaviruses drug repurposing studies are currently approaching 3,000 citations on Google Scholar (Gordon et al., 2020). The percentage of highly cited studies and hot research topics related to novel coronaviruses is also a good indication that the method has made an indelible contribution to the study of novel coronaviruses and similar infectious diseases. Auxiliary technology for the experimental screening of traditional drugs is also developing (Langhans, 2018), which also promotes drug repositioning or other drug development processes. Furthermore, the high-quality results of Elfiky, AA, a scientist from Cairo University, Egypt (Elfiky, 2020), suggest that relevant research in some economically underdeveloped countries may reach top levels worldwide due to the return or affiliation of some prominent scientists.

## 5 Conclusion

For this research, the literature on drug repositioning research published in the SCI-E and SSCI sections of WOS core journals from 2010 to 2020 was analyzed based on bibliometrics and DDA software. This area has been of interest to scientists since the end of the 20th century and entered a period of rapid growth in 2019, with the peak far from being reached. Using bibliometrics as a tool, the United States has become a world leader in terms of the number of submissions, number of high-quality studies, funding support, strength of research institutions, and number of top scholars, followed by China and the United Kingdom, where more research is being performed in this area. As a method of drug discovery, drug repurposing is closely related to the development of various biomedical disciplines, and computer-related disciplinary methods, such as mathematical computational biology and computer science, have taken an important place in the research of this field in the last decade. The authors’ keyword analysis suggests that research in the field of the novel coronavirus will remain valuable until the associated pandemic is completely contained. Virtual screening, molecular docking, machine learning, and other related technical fields still need long-term development to achieve efficient and accurate repositioning of drugs (Kumar et al., 2019). Precision medicine, combined with drug repositioning, is the most promising direction for the future. In conclusion, drug repositioning can help to treat more diseases, such as drug resistance, poor drug selectivity, and limited therapeutic options.

This study may help some scholars with an initial interest in drug repositioning-related research to gain a concise and rapid understanding of the current state of global research, as well as offer some relevant information to institutions or groups seeking collaboration.

## 6 Limitations

It is worth noting that this study has some biases and limitations. First, there are still some issues with the publications included in the study based on subject terms: 1) some relevant publications that do not use the search formula in this study may have been excluded from this study and 2) there may also be a small number of articles whose use of some of the aforementioned search terms deviates significantly from the general understanding; yet, such publications are included in this study. Second, some extraneous factors distort the credibility of the bibliometric statistics. 1) When analyzing the keywords of publications, some publications are excluded from the statistical analysis because they do not list author keywords (e.g., (Gordon et al., 2020)). 2) Excessive self-citation by some authors (Haghighat and Hayatdavoudi, 2021) inflates the actual level of interest in the publication. 3) for a publication, when an author submits more than one institution’s address information, this publication is counted as research results by each institution. Finally, in future work, patents from the WOS database associated with the topic of drug repositioning will be analyzed to provide another perspective on the situation of the topic in terms of applications and technological innovations.
